# Mosquito Shield™, a transfluthrin passive emanator, protects against pyrethroid-resistant *Anopheles gambiae* sensu lato in central Benin

**DOI:** 10.1186/s12936-024-05043-5

**Published:** 2024-07-31

**Authors:** Augustin Fongnikin, Juniace Ahoga, Boris Ndombidje, Corneille Hueha, Esperantos de Souza, Ruth Oti-Tossou, Renaud Govoetchan, Corine Ngufor

**Affiliations:** 1 Centre de Recherches Entomologiques de Cotonou (CREC), Cotonou, Benin; 2 Pan African Malaria Vector Research Consortium (PAMVERC), Cotonou, Benin; 3 African Institute for Research in Infectious Diseases (AIRID), Cotonou, Benin; 4 London School of Hygiene and Tropical Medicine (LSHTM), London, WC1E 7HT UK

## Abstract

**Background:**

Spatial repellents can provide personal and household protection against biting vector mosquitoes by volatizing repellents into the air within a given area. Mosquito Shield™ is a transfluthrin passive emanator undergoing evaluation for malaria control. Studies evaluating its entomological impact against different local malaria vector populations would help guide its deployment in endemic countries.

**Methods:**

A two-arm single-blinded small-scale household randomised entomological trial was conducted to assess the impact of Mosquito Shield™ on the human landing rate of wild pyrethroid-resistant *Anopheles gambiae *sensu lato (*s.l*.) vector mosquitoes in houses in the Ganhoua village of the Zakpota District of central Benin. From a total of 30 houses, 15 were randomly allocated to receive Mosquito Shield™, while the remainder received a placebo product. The trial lasted through the life of the Mosquito Shield™ product (32 days). Mosquito sampling was performed by human landing catches at baseline and at 6 timepoints post-intervention (days 0–1, 7–8, 14–15, 21–22, 28–29 and 31–32). Collections were performed for 2 nights at each sampling time point. WHO cylinder bioassays were conducted during the trial with F1 *An. gambiae s.l.* mosquitoes that emerged from larvae from the study area to assess the intensity of resistance to pyrethroids in the wild vector population.

**Results:**

The vector population in the study area showed a high intensity of resistance to pyrethroids. Baseline *An. gambiae s.l.* human landing rates were similar in houses in both study arms before product application (11.53/person/night vs 11.67/person/night, p > 0.05). A total of 5736 mosquitoes were collected in the placebo control arm and 3862 in the Mosquito Shield™ arm post-intervention. Overall *An. gambiae s.l.* post-intervention human landing rates were significantly lower in houses in the Mosquito Shield™ arm (18.13/person/night) compared to the houses in the placebo control arm (26.84/person/night, IRR = 0.658, p < 0.001). Over the lifespan of the product, Mosquito Shield™ provided a significant protective efficacy of 34.2% (22.1–44.4%, p < 0.001) against wild pyrethroid-resistant *An. gambiae s.l.* vectors compared to the placebo. Human landing rates of other nuisance vector mosquito species (*Culex* and *Mansonia*) were also reduced in houses treated with Mosquito Shield™ compared to the placebo.

**Conclusion:**

Mosquito Shield™, a transfluthrin passive emanator, provided significant protection against pyrethroid-resistant malaria vectors to households in Benin. The spatial repellent shows potential to reduce malaria transmission by pyrethroid-resistant *An. gambiae s.l.* vector mosquitoes and cover gaps in malaria control when deployed to complement existing vector control interventions.

## Background

Vector control through the large-scale deployment of insecticide-treated nets (ITNs) and indoor residual spraying (IRS) contributed substantially to the remarkable reductions in malaria burden between 2000 and 2015 [1]. Global progress against malaria has unfortunately stalled in recent years and is expected to go further off course if no additional measures are implemented [2]. This stalled progress has been attributed to several factors including the development of vector resistance to the insecticides used on ITNs and IRS, poor access and durability of ITNs and reduced funding for malaria control [2]. This highlights the need to both strengthen the impact of existing tools and bring to market new cost-effective vector control interventions to fill gaps in protection and facilitate advancements towards global malaria elimination targets.

Spatial repellents are airborne repellent compounds that alter mosquito behaviours inducing movement away from a chemical stimulus and interfering with host detection and feeding [3]. By disrupting the mosquito’s host-seeking behaviour when volatilized into the air within a given area, they reduce human vector contact and thus provide personal and household protection potentially reducing disease transmission [3–5]. Commercial spatial repellent products, such as mosquito coils, and electrical plug-ins are widely available for protection from mosquito bites. However, these products require a heat source to volatize the active ingredient (AI) and disperse adequate concentrations into the target area resulting in poor user compliance and health risks associated with the smoke generated by burning [6–8]. Passive emanator spatial repellents were developed to provide a volatile concentration of the repellent active ingredient at room temperature from a point source using only natural airflow thus requiring little to no compliance from the user [9]. Their efficacy against *Aedes* mosquito vectors of diseases such as dengue, chikungunya and Zika has been demonstrated in multiple studies [9, 10].

Mosquito Shield™, a transfluthrin passive emanator developed by SC Johnson & Son Inc, is a spatial repellent product designed to be easy to use with minimal handling, which may help to increase user compliance and acceptability. It can be hung in semi-enclosed and enclosed spaces to continuously protect against bites from mosquitoes. The emanator consists of a multilayer plastic film pre-treated with 110 mg of transfluthrin which passively emanates using natural airflow to protect people from mosquitoes in a specific area. Previous studies in Peru demonstrated the potential of an earlier prototype of Mosquito Shield™ to provide substantial reductions in human-vector contact and *Aedes*-borne viral disease transmission [11]. A cluster randomized controlled trial (RCT) in Indonesia showed a 60% protective efficacy against malaria infection in moderate to high-risk clusters that received the product [12]. Further RCTs to determine the public health value of Mosquito Shield™ as part of the required evidence for the endorsement of the intervention for malaria control by the World Health Organization (WHO), are ongoing in Kenya [13] and Mali [14]. In addition to RCTs, small-scale entomological studies investigating the impact of spatial repellent passive emanators against local malaria vectors may help guide their deployment. Several semi-field entomological trials conducted in East Africa have demonstrated the capacity of transfluthrin-based spatial repellent products including Mosquito Shield™ to protect humans from malaria mosquito bites [15–17]. There is however little to no evidence of their entomological performance in West Africa where local vectors have historically exhibited higher levels of pyrethroid resistance [18].

A small-scale household randomised trial was thus conducted to assess the entomological efficacy of Mosquito Shield™ in households in the Zakpota District of central Benin where the local vector population shows a high intensity of resistance to pyrethroids. Thirty households recruited from the study area were randomized to receive Mosquito Shield™ or a placebo product and performance was assessed in terms of the reduction in landing rates of wild vector mosquitoes on humans in Mosquito Shield™ treated households compared to the placebo-treated households.

## Methods

### Study area

The study was performed in the Ganhoua village situated in the Za-Kpota District (7.2384° N, 2.2040° E) of the Zou Department of Benin. Results from a recent entomological survey conducted in the Za-Kpota District showed that the main vector, *Anopheles gambiae *sensu lato (*s.l*.) consists of ~ 45% *Anopheles coluzii* and ~ 55% *An*. *gambiae *sensu stricto (*s.s*.) and is highly resistant to pyrethroids mediated by high levels of *kdr* (> 80%) and overexpressed P450 enzymes [19].

### Study design and sample size considerations

This was a two-armed single-blinded small-scale household randomized entomological trial with houses as units of observation. The evaluation was performed in a total of 30 houses; 15 were randomly allocated to receive the Mosquito Shield™ product and the remainder received a placebo that was similar to the product but did not contain the active ingredient. Based on human landing rates of the main malaria vector per house per night observed in a previous study in the study area, with a total of 12 collection nights in each house through the life of the product, the study design had > 80% power to detect a 25% reduction in human landing rates with Mosquito Shield™. After the recruitment of households, a baseline survey was conducted to assess household characteristics, mosquito species composition and human landing rates in each house. Treatments were then applied, and houses were assessed for the impact of the product on human landing rates of the major malaria vector at specific time points throughout the life of the product. Householders and mosquito collectors were blinded to the intervention applied in each house.

### Recruitment and allocation of participating households

**Fig. 1:**
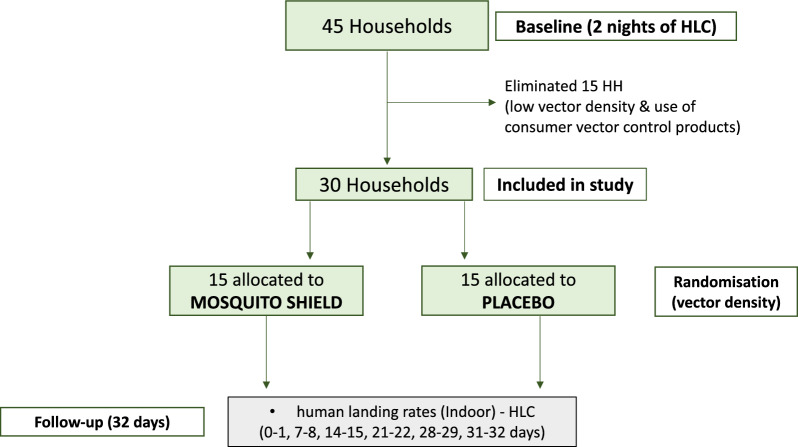
Study flowchart

Approximately forty-five (45) households were initially recruited at baseline. Houses were recruited within a 2.5 km transect of the village and were within 10–15 min walking distance from each other. Households with pregnant and/or nursing mothers were excluded for safeguarding reasons. The study details and frequency of sampling were explained to householders by the study team in their local language with support from community health workers. Following recruitment, a baseline survey was conducted to collect data on the construction characteristics, presence of ITNs and frequency of use of vector control products in each household. To help guide the deployment of the product, measurements were taken of each room in the recruited household. Baseline entomological indices for malaria vectors including species composition and human landing rates, were collected via human landing catches (HLC). Out of the 45 households recruited at baseline, thirty were included in the evaluation (Fig. 1). Houses showing very low vector mosquito landing rates and high use of consumer vector control products (coils, sprays, repellents) at baseline were excluded. Households included in the evaluation also agreed to not use consumer vector control products during the study. Selected households were then randomly allocated to each study arm based on baseline *An. gambiae s.l.* human landing rates. Randomization was performed multiple times to ensure that both study arms were similar in terms of *An. gambiae s.l.* human landing rates at baseline.

### Installation of Mosquito Shield™ products

**Fig. 2:**
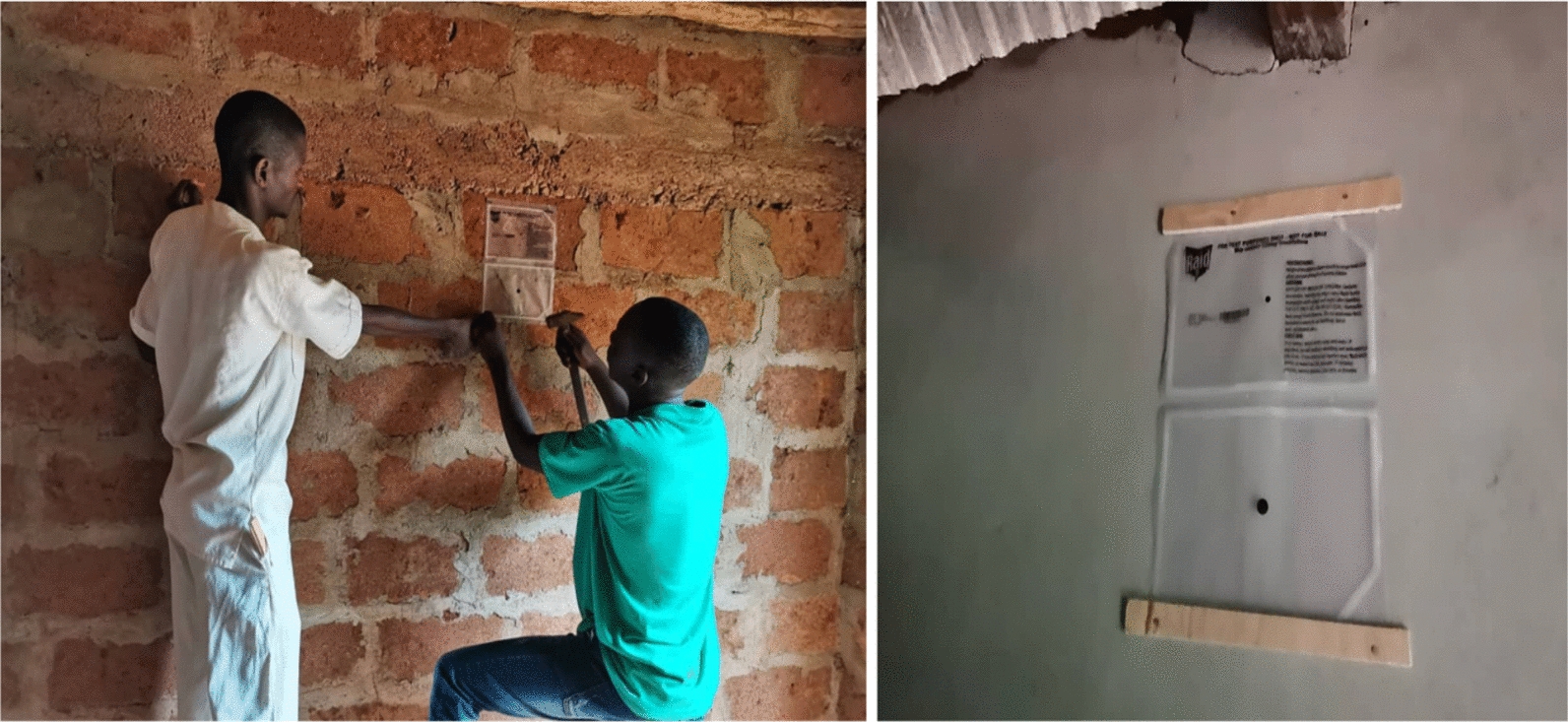
Installation of Mosquito Shield™ products on home walls

All rooms in study houses were between 9 m^2^ and 18 m^2^ in size thus requiring 4 Mosquito Shield™ products per room per house (1 per wall) as indicated by the manufacturer’s instructions. Installation was done by the study team in each study house. Products were fixed on walls using nails and small pieces of wood and were placed at approximately two-thirds of the wall height from the floor (Fig. 2). Placebo products were installed using the same method. A total of 140 Mosquito Shield™ and 132 placebo products were installed in the houses included in the study.

### Mosquito sampling and processing

The trial was performed from April to May 2022 and lasted for the duration of the efficacy of the Mosquito Shield™ product (32 days). Mosquitoes were sampled in recruited houses once at baseline and 6 times within the life of the product (0–1, 7–8, 14–15, 21–22, 28–29, 31–32 days) post-treatment application, using HLC. Sampling was done for 2 consecutive nights at each round of sampling in one dedicated room in each house agreed upon in consultation with the head of the household. Consenting human volunteer mosquito collectors working in pairs collected mosquitoes landing on their legs overnight from 7 pm to 7 am in each house on each sampling day. Collections were done in two shifts each night (7 pm to 1 am and 1 am to 7 am). The collectors sat on chairs with their lower limbs exposed and collected all mosquitoes, which landed on them using sucking tubes. To characterize *Anopheles* biting time behaviour, collections were recorded per hour. To control human attractiveness to mosquitoes, for each collection round, human collector pairs were rotated between houses in the intervention arm and the placebo arm on successive nights.

In the morning, all collected mosquitoes were transported to the laboratory for morphological identification to species level using appropriate taxonomic keys. The date of collection, house identification number and species were recorded for each mosquito. The key outcome measure used to determine the efficacy of Mosquito Shield™ in the study houses was the protective efficacy defined as the percentage reduction in human landing rates of malaria vector mosquitoes collected by HLC in households in the treated arm compared to the placebo arm.

### Data analysis

Differences in the numbers of female *An. gambiae s.l.* landing per person per household were analysed using mixed effects negative binomial regression in STATA version 17 with treatments, collection timepoint and households added as fixed/random effects. Vector incidence rate ratios (IRR) between the Mosquito Shield™ and placebo arms and 95% confidence intervals were calculated for the entire duration of the study and for each sampling timepoint.

Overall efficacy was expressed in terms of the protective efficacy (PE) against mosquito landing rates in the Mosquito Shield™ arm relative to the control placebo arm which was calculated as follows:

$PE=(1-IRR)\times 100$ where IRR is the incidence rate ratio in the Mosquito Shield™ group compared to the placebo group.

### Ethical considerations

The study received ethical approval from the ethics review committee of the Ministry of Health in Benin (CNERS, No. 54). Informed consent was obtained from the head of each participating household and mosquito collectors before they participated in the study (Appendix 3). The consent forms and participant information sheets were explained to them in their local language. An impartial witness was used when participants could not read or write. Mosquito collectors were offered chemoprophylaxis during the study. A study nurse was available to examine any cases of fever and any collectors found positive for malaria, were treated free of charge during the study and up to 4 weeks after the end of the study. Participants were free to withdraw from the study at any time.

### Susceptibility tests

To determine the frequency of insecticide resistance in the wild vector population of the study area during the trial, WHO cylinder bioassays were performed on 2 to 5 days old adult F1 female mosquitoes emerging from larvae collected from breeding sites around the study houses. Approximately 100 female mosquitoes per insecticide were exposed for 1 h in batches of 25 to alpha-cypermethrin 0.05% and permethrin 0.75% and to filter papers treated 5× and 10× the diagnostic doses of these insecticides. Knockdown was recorded after 1 h and mortality after a 24 h holding period.

## Results

### Baseline characteristics of participating households

The average number of inhabitants per household among the 30 households included in the study was 2 and this was similar between households in both study arms (Table 1). All households owned at least one LLIN. The proportion of people who reported sleeping under nets every night was 80%. Most nets were PermaNet® 2.0 (13/30) and Yorkool® (14/30). Overall, most houses were made of either cement or a mixture of mud and cement (27/30). Two households reported using consumer vector control products on some nights. *An. gambiae s.l*, was the most abundant mosquito species in the study houses at baseline and represented 73% of the collection. Other species collected in lower densities were *Mansonia africana* (18%) and *Culex quinquefasciatus* (8%).

**Table 1:** Household characteristics

	Placebo	Mosquito Shield™	Total	
	N	N	N	(%)
Indicators				
Total N of households	15	15	30	–
Total N of people	51	40	91	–
Number of rooms for sleeping	26	20	46	–
Mean number of persons per house	2	2	2	
Type of housing				
Mud	3	4	7	23.33
Cement	8	5	13	43.33
Mud + Cement	4	6	10	33.33
LLINs				
PermaNet 2.0	5	8	13	43.33
Yorkool	8	6	14	46.66
Other LLINs	1	2	3	10
Use of LLINs				
Every night (7 nights)	12	12	24	80
Use of consumer vector control products				
Every night (7 nights)	0	0	0	0
Most nights (5–6)	0	0	0	0
Some night (1–4 nights)	1	1	2	6.66
Not used last week	1	1	2	6.66

### Species composition and overall mosquito landing rates post-intervention

A total of 5736 female mosquitoes were collected in the placebo arm and 3862 female mosquitoes in the Mosquito Shield™ arm post-intervention giving an overall reduction in mosquito landing rates of 32.67% (Table 2). The largest proportion of mosquito species collected were *An. gambiae s.l.* followed by *Culex* spp and *Mansonia* spp.. Small numbers of secondary malaria vectors (*Anopheles pharoensis* and *Anopheles ziemani*) were also collected. Species composition in terms of the proportion of each species, was generally similar across both study arms. Overall, for each mosquito genera, lower numbers were collected in houses in the Mosquito Shield™ arm compared to the placebo arm (Table 2) and these differences were significant for *Anopheles*, *Culex* and *Mansonia* mosquitoes (26.57–35.20%, p < 0.05). Reductions in *Aedes aegypti* were observed though the numbers collected were too few (13).

**Table 2:** Total numbers and species composition of mosquitoes collected by HLC per study arm

Species	Placebo		Mosquito Shield		Reduction in numbers collected (%)
	N	%	N	%	
*Anopheles gambiae s.l*	4831	84.22	3263	84.49	32.46
*Anopheles pharoensis*	93	1.62	49	1.27	47.31
*Anopheles ziemani*	12	0.21	1	0.03	91.67
*Aedes aegypti*	13	0.23	2	0.05	84.62
*Culex species*	358	6.24	232	6.01	35.20
*Mansonia species*	429	7.48	315	8.16	26.57
*Total*	5736	100	3862	100	32.67

### Reduction in human landing rates of *An. gambiae s.l.*

**Fig. 3:**
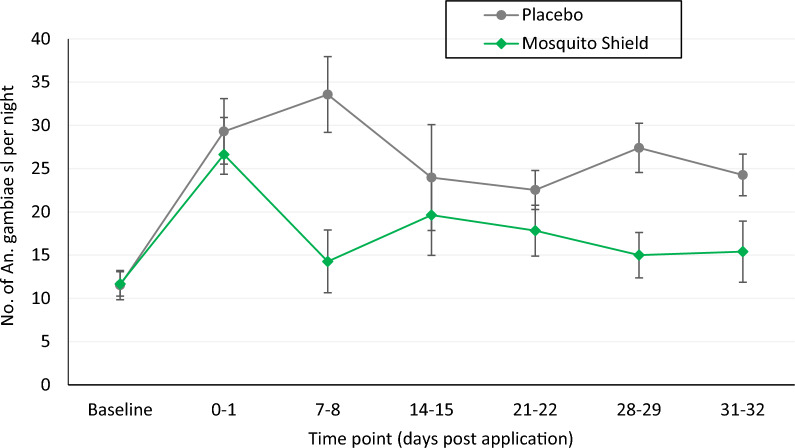
*An. gambiae* sl. mosquitoes human landing rate per house per night per study arm

The *An. gambiae s.l.* landing rates in both study arms at each time point and reductions observed with Mosquito Shield™ relative to the placebo control arm are shown in Table 3 and Fig.3. Landing rates were lower at baseline compared to post-intervention time points and this can be attributed to local changes in vector density over time. Landing rates were generally lower in houses in the Mosquito Shield™ arm compared to the placebo control arm at all 6 post-intervention sampling timepoints and these differences were significant at most time points (P < 0.05). Overall, mosquito landing was significantly lower in houses with Mosquito Shield™ (18.13 bites per person per night) compared to the placebo control (26.84 bites per person per night, IRR = 0.658, P < 0.001). The protective efficacy of Mosquito Shield™ was lowest in the first round of collection (9.1% at 0–1-day, p = 0.335) but increased in the subsequent time points and ranged from 18.1% to 59%. This was probably due to the product taking some time to build up the volatile AI in the treated houses.

**Table 3:** Human landing rates and protective efficacy of Mosquito Shield™ against wild *An. gambiae sl* in households in Ganhoua village, Zakpota sub-district, Benin

Time point	Arm	Total collected	Person nights	HBR	IRR (95% CI)	% Protective efficacy (95% CI)	p value
Baseline	Placebo	346	30	11.53	1.012 (0.834–1.226)	n/a	0.907
	Mosquito Shield	350	30	11.67			
0–1 days	Placebo	879	30	29.30	0.909 (0.749–1.104)	9.1 (0–25.1)	0.335
	Mosquito Shield	799	30	26.63			
7–8 days	Placebo	1007	30	33.57	0.410 (0.309–0.544)	59 (45.6–69.1)	< 0.001
	Mosquito Shield	428	30	14.27			
14–15 days	Placebo	719	30	23.97	0.819 (0.535–1.255)	18.1 (0–46.5)	0.359
	Mosquito Shield	589	30	19.63			
21–22 days	Placebo	676	30	22.53	0.779 (0.654–0.927)	22.1 (7.3–54.8)	0.005
	Mosquito Shield	535	30	17.83			
29–30 days	Placebo	822	30	27.40	0.540 (0.452–0.644)	46 (35.6–54.8)	< 0.001
	Mosquito Shield	450	30	15.00			
31–32 days	Placebo	728	30	24.27	0.599 (0.463–0.774)	40.1 (22.6–53.7)	< 0.001
	Mosquito Shield	462	30	15.40			
Total (post intervention)	Placebo	4831	180	26.84	0.658 (0.556–0.779)	34.2 (22.1–44.4)	< 0.001
	Mosquito Shield	3263	180	18.13			

**Fig. 4:**
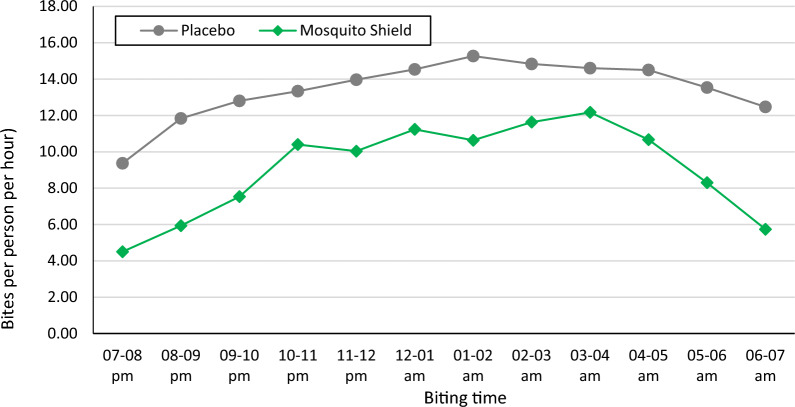
Overall human biting rate of *Anopheles gambiae* sl per hour in houses treated with Mosquito Shield™ compared to a placebo

Mosquito Shield™ provided a significant overall protective efficacy of 34.2% (22.1–44.4%, p < 0.001) post-intervention. Hourly biting rates of *An. gambiae sl* were also consistently higher in the placebo control arm compared to the Mosquito Shield™ arm at all times of the night (Fig. 4). A larger reduction in mosquito biting was observed in the early morning hours (4:00 am to 7:00 am) with Mosquito Shield™ compared to the placebo (Fig. 4).

### Susceptibility test results

**Fig. 5:**
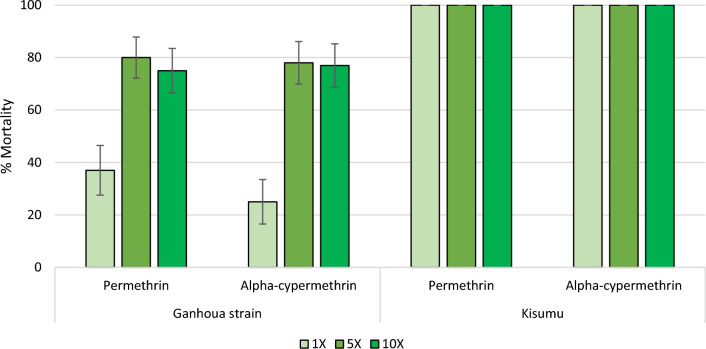
Mortality rates of *An. gambiae sl* to 0.05%, 0.25% and 0.5% alpha-cypermethrin, 0.75%, 3.75% and 7.5% permethrin. The error bars indicate the 95% confidence intervals

Mortality rates of the susceptible laboratory-maintained *An. gambiae s.s*. Kisumu strain after exposure to permethrin and alpha-cypermethrin treated papers in WHO cylinder bioassays were both 100%. With the wild *An. gambiae s.l.* from the study area (Ganhoua), mortality rates were 37% at 1X, 80% at 5X and 75% for permethrin and 25% at 1X, 78% at 5X and 77% at 10X for alpha-cypermethrin (Fig. 5) showing that the wild strain had a high intensity of resistance to both pyrethroid insecticides.

## Discussion

The purpose of this study was to evaluate the impact of Mosquito Shield™, a transfluthrin passive emanator on human landing rates of wild free-flying high-intensity pyrethroid-resistant *An. gambiae s.l.* in houses in the Ganhoua village in the Zakpota district of central Benin. The data showed a significant protective effect of 34.2% against malaria mosquitoes in Mosquito Shield™ treated houses relative to houses treated with a placebo. The intervention remained efficacious through its 1-month product span. The findings demonstrate the impact of a transfluthrin passive emanator against local malaria vectors in Benin for the first time and corroborate multiple studies that have shown the potential of spatial repellents to protect humans from vector mosquitoes in other countries [9, 15, 17].

The levels of protective efficacy achieved with Mosquito Shield™ in this study (34.2%) were nevertheless lower than what was observed against *Anopheles arabiensis* (70% protective efficacy) in a recent experimental hut trial in Tanzania, East Africa [17]. The difference in outcome between both studies could be attributed to higher intensities of pyrethroid resistance in the *An. gambiae s.l.* population in the Benin study and/or to inherent differences in vector species. The *An. gambiae s.l.* vector population in Benin is highly anthropophagic, feeding almost entirely on humans indoors [20], while *An arabiensis* tends to be more zoophilic and is less attracted to humans [21, 22]. Susceptibility bioassays showed that the Benin vector population had a high intensity of resistance to pyrethroids with mosquito mortality remaining below 80% even at doses 10 times the diagnostic dose of permethrin and alpha-cypermethrin. This high intensity of resistance to the pyrethroids coupled with the higher local vector attractiveness to humans may, therefore, have reduced the capacity of the transfluthrin passive emanator to sufficiently modify the mosquitoes’ host-seeking behaviour in the Benin study and induce greater levels of protection.

While it is unclear how reductions in mosquito landing rates will impact malaria incidence and prevalence, traditional malaria transmission models indicate that reducing human landing rates and thus human vector contact even at the levels demonstrated in this study can have major effects on the vectorial capacity of a vector population [23]. The findings therefore show the potential of the Mosquito Shield™ to reduce malaria transmission by pyrethroid-resistant *An. gambiae s,l,* vectors to individuals in treated households in a West African setting where vectors historically exhibit high intensities of pyrethroid resistance [18]. Results from the ongoing RCT of Mosquito Shield™ in Mali [14] may further elucidate the epidemiological impact of the intervention on clinical malaria when applied at a community scale in the region.

The Mosquito Shield™ product was designed to last approximately 1 month, and, in this study, we demonstrated its efficacy for this period. However, malaria transmission seasons in endemic countries typically last several months hence it is expected that under operational conditions, the Mosquito Shield™ product will have to be replaced multiple times in houses to cover the entire transmission season. While installation was relatively easy for the study team, requiring less than 15 min per house, the process can be very demanding for householders if it must be done too frequently. To overcome this challenge, an advanced longer-lasting version of the transfluthrin passive emanators has been developed by the manufacturer and is undergoing evaluation in semi-field studies across Africa.

Mosquito Shield™ induced substantial reductions in human landing rates of *Culex* and *Mansonia* (26–35%) mosquitoes, vectors of human filariasis, in treated houses compared to the placebo. Reductions in densities of such nuisance mosquitoes have been reported in multiple small-scale trials of transfluthrin spatial repellents [16, 24]. Protection from nuisance mosquitoes is usually associated with an increased uptake of malaria vector control interventions. This finding may, therefore, have positive implications for the acceptability of Mosquito Shield™ to householders. Further studies investigating user acceptance at both household and community levels are advisable.

This study evaluated the entomological impact of transfluthrin passive emanators in households which had pyrethroid-only nets in them. Following WHO’s recent endorsement of dual active ingredient nets, [25], many endemic countries are replacing pyrethroid-only nets with these new more effective nets. Passive emanators will therefore likely be deployed against a background of high coverage with dual AI nets. Studies investigating their potential to complement dual AI nets may help guide local deployment strategies.

## Conclusion

This study demonstrated a 34.2% protective efficacy of Mosquito Shield™, a transfluthrin passive emanator, against a high-intensity wild pyrethroid-resistant malaria vector population when applied in houses in Benin. Mosquito Shield™ remained protective throughout its product life of 30 days. The passive emanator shows potential to improve the control of malaria transmitted by pyrethroid-resistant *An. gambiae s,l,* vector mosquitoes and to help cover gaps in malaria control that may exist with core vector control tools.

## Data Availability

The datasets used and/or analysed during the current study are available from the corresponding authors on reasonable request.

## Abbreviations

ITN: Insecticide-treated nets
IRS: Indoor residual spraying
WHO: World Health Organization
AI: Active ingredient
HLC: Human landing catches
HBR: Human biting rate
IRR: Incidence rate ratio
RCT: Randomized controlled trials
CREC: Centre de Recherche Entomologique de Cotonou
LSHTM: London School of Hygiene & Tropical Medicine
PAMVERC: Pan African Malaria Vector Research Consortium
AIRID: African Institute for Research in Infectious Diseases

## References

[1] Bhatt S, Weiss D, Cameron E, Bisanzio D, Mappin B, Dalrymple U, et al. The effect of malaria control on *Plasmodium falciparum* in Africa between 2000 and 2015. Nature. 2015;526:207–11. 26375008 10.1038/nature15535 PMC4820050

[2] WHO. World Malaria report. Geneva: World Health Organization; 2023.

[3] WHO. Guidelines for efficacy testing of spatial repellents. Geneva, World Health Organization, 2013, https://www.who.int/publications/i/item/9789241505024.

[4] Achee NL, Bangs MJ, Farlow R, Killeen GF, Lindsay S, Logan JG, Moore SJ, et al. Spatial repellents: from discovery and development to evidence-based validation. Malar J. 2012;11:164. 22583679 10.1186/1475-2875-11-164 PMC3453515

[5] Achee NL, Perkins TA, Moore SM, Liu F, Sagara I, Van Hulle S, et al. Spatial repellents: the current roadmap to global recommendation of spatial repellents for public health use. Curr Res Parasitol Vector Borne Dis. 2023;3: 100107. 36590345 10.1016/j.crpvbd.2022.100107 PMC9801085

[6] Hogarh JN, Antwi-Agyei P, Obiri-Danso K. Application of mosquito repellent coils and associated self-reported health issues in Ghana. Malar J. 2016;15:61. 26847206 10.1186/s12936-016-1126-8 PMC4743129

[7] Liu W, Zhang J, Hashim JH, Jalaludin J, Hashim Z, Goldstein BD. Mosquito coil emissions and health implications. Environ Health Perspect. 2003;111:1454–60. 12948883 10.1289/ehp.6286 PMC1241646

[8] Lawrance CE, Croft AM. Do mosquito coils prevent malaria? A systematic review of trials. J Travel Med. 2004;11:92–6. 15109473 10.2310/7060.2004.17015

[9] Devine GJ, Vazquez-Prokopec GM, Bibiano-Marin W, Pavia-Ruz N, Che-Mendoza A, Medina-Barreiro A, et al. The entomological impact of passive metofluthrin emanators against indoor *Aedes aegypti*: a randomized field trial. PLoS Negl Trop Dis. 2021;15: e0009036. 33497375 10.1371/journal.pntd.0009036 PMC7864418

[10] Buhagiar TS, Devine GJ, Ritchie SA. Metofluthrin: investigations into the use of a volatile spatial pyrethroid in a global spread of dengue, chikungunya and Zika viruses. Parasit Vectors. 2017;10:270. 28558804 10.1186/s13071-017-2219-0 PMC5450184

[11] Morrison AC, Reiner RC Jr, Elson WH, Astete H, Guevara C, Del Aguila C, et al. Efficacy of a spatial repellent for control of *Aedes*-borne virus transmission: a cluster-randomized trial in Iquitos. Peru Proc Natl Acad Sci USA. 2022;119: e2118283119. 35737833 10.1073/pnas.2118283119 PMC9245620

[12] Syafruddin D, Asih PBS, Rozi IE, Permana DH, Nur Hidayati AP, Syahrani L, et al. Efficacy of a spatial repellent for control of malaria in Indonesia: a cluster-randomized controlled trial. Am J Trop Med Hyg. 2020;103:344–58. 32431275 10.4269/ajtmh.19-0554 PMC7356406

[13] Ochomo EO, Gimnig JE, Bhattarai A, Samuels AM, Kariuki S, Okello G, et al. Evaluation of the protective efficacy of a spatial repellent to reduce malaria incidence in children in western Kenya compared to placebo: study protocol for a cluster-randomized double-blinded control trial (the AEGIS program). Trials. 2022;23:260. 35382858 10.1186/s13063-022-06196-x PMC8980512

[14] Van Hulle S, Sagara I, Mbodji M, Nana GI, Coulibaly M, Dicko A, et al. Evaluation of the protective efficacy of a spatial repellent to reduce malaria incidence in children in Mali compared to placebo: study protocol for a cluster-randomized double-blinded control trial (the AEGIS program). Trials. 2022;23:259. 35382856 10.1186/s13063-022-06197-w PMC8980511

[15] Ogoma SB, Ngonyani H, Simfukwe ET, Mseka A, Moore J, Killeen GF. Spatial repellency of transfluthrin-treated hessian strips against laboratory-reared *Anopheles arabiensis* mosquitoes in a semi-field tunnel cage. Parasit Vectors. 2012;5:54. 22433128 10.1186/1756-3305-5-54 PMC3338372

[16] Ogoma SB, Mmando AS, Swai JK, Horstmann S, Malone D, Killeen GF. A low technology emanator treated with the volatile pyrethroid transfluthrin confers long term protection against outdoor biting vectors of lymphatic filariasis, arboviruses and malaria. PLoS Negl Trop Dis. 2017;11: e0005455. 28388682 10.1371/journal.pntd.0005455 PMC5384659

[17] Swai JK, Soto AC, Ntabaliba WS, Kibondo UA, Ngonyani HA, Mseka AP, et al. Efficacy of the spatial repellent product Mosquito Shield™ against wild pyrethroid-resistant *Anopheles arabiensis* in south-eastern Tanzania. Malar J. 2023;22:249. 37649032 10.1186/s12936-023-04674-4 PMC10466708

[18] Hancock PA, Hendriks CJM, Tangena JA, Gibson H, Hemingway J, Coleman M, et al. Mapping trends in insecticide resistance phenotypes in African malaria vectors. PLoS Biol. 2020;18: e3000633. 32584814 10.1371/journal.pbio.3000633 PMC7316233

[19] Ngufor C, Govoetchan R, Fongnikin A, Hueha C, Ahoga J, Syme T, et al. Community evaluation of VECTRON™ T500, a broflanilide insecticide, for indoor residual spraying for malaria vector control in central Benin; a two arm non-inferiority cluster randomised trial. Sci Rep. 2023;13:17852. 37857762 10.1038/s41598-023-45047-w PMC10587144

[20] Akogbéto MC, Salako AS, Dagnon F, Aïkpon R, Kouletio M, Sovi A, et al. Blood feeding behaviour comparison and contribution of *Anopheles coluzzii* and *Anopheles gambiae*, two sibling species living in sympatry, to malaria transmission in Alibori and Donga region, northern Benin, West Africa. Malar J. 2018;17:307. 30134912 10.1186/s12936-018-2452-9 PMC6106899

[21] Mlacha YP, Chaki PP, Muhili A, Massue DJ, Tanner M, et al. Reduced human-biting preferences of the African malaria vectors *Anopheles arabiensis* and *Anopheles gambiae* in an urban context: controlled, competitive host-preference experiments in Tanzania. Malar J. 2020;19:418. 33218346 10.1186/s12936-020-03495-z PMC7678205

[22] Takken W, Verhulst NO. Host preferences of blood-feeding mosquitoes. Annu Rev Entomol. 2013;58:433–53. 23020619 10.1146/annurev-ento-120811-153618

[23] Macdonald G. Epidemiological basis of malaria control. Bull World Health Organ. 1956;15:613–26. 13404439 PMC2538278

[24] McMillan BE, Britch SC, Golden FV, Aldridge RL, Moreno BJ, Bayer BE, et al. Assessing transfluthrin mortality against *Aedes aegypti* and *Culex quinquefasciatus* inside and outside US military tents in a northern Florida environment. Curr Res Parasitol Vector Borne Dis. 2022;2: 100067. 36589865 10.1016/j.crpvbd.2021.100067 PMC9795342

[25] WHO. Guidelines for malaria vector control. Geneva: World Health Organization; 2023. 30844152

